# Prognostic utility of immune biomarkers for identifying individuals at risk of unfavourable treatment outcomes in pulmonary tuberculosis

**DOI:** 10.3389/fimmu.2026.1928573

**Published:** 2026-10-05

**Authors:** Agalya Vanamudhu, Vijay Viswanathan, Guruprashath Mohan, Nivedha Krishna Rao, Kannan Thiruvengadam, Syed Hissar, Bhavani Permal Kannabiran, Subash Babu, Hardy Kornfeld, Nathella Pavan Kumar

**Affiliations:** 1 Prof. M. Viswanathan Diabetes Research Center, Chennai, India; 2 Division of TB Prevention and Elimination Research, ICMR- National Institute for Research in Tuberculosis, Chennai, India; 3 National Institutes of Health-National Institute for Research in Tuberculosis (NIRT)- International Center for Excellence in Research, Chennai, India; 4 Laboratory of Parasitic Diseases (LPD), of National Institute of Allergy and Infectious Diseases (NIAID), National Institutes of Health (NIH), Bethesda, MD, United States; 5 UMass Chan Medical School, Worcester, MA, United States

**Keywords:** biomarkers, chemokines, cytokines, MMPs, tuberculosis

## Abstract

**Introduction:**

Tuberculosis remains a major global health challenge, with many patients expe- riencing unfavourable treatment outcomes despite standard therapy. Host immune biomarkers, including cytokines, chemokines, and matrix metalloproteinases (MMPs), have shown promise in predicting disease severity and treatment response, but require validation across independent cohorts.

**Methods:**

We validated previously identified immune biomarkers using baseline plasma samples from a nested case–control cohort within the RePORT India consortium (cases: n=74; controls: n=161). Cytokines (IL-2, IFN-γ, TNF-α, IL-6, IL-1β, IL-10, IL-17A, and GM-CSF), chemokines (CCL2, CCL3, CCL4, CXCL1, CXCL8, and CXCL10), and matrix metalloproteinases (MMP-1, MMP-2, MMP-7, and MMP-9) were quantified using multiplex ELISA on the Luminex platform. Statistical analyses included group comparisons, ROC analysis, logistic regression, random forest modelling, princi- pal component analysis, and stability selection to identify optimal biomarker combinations.

**Results:**

Elevated levels of IL-2, IFN-γ, TNF-α, IL-6, CCL2, CXCL8, CXCL10, MMP-2, MMP-7, and MMP-9 were associated with unfavourable out- comes. IL-2, CXCL8, CCL2, and MMP-2 showed strong individual predictive performance (AUC ≥0.80). Multivariable analysis identified CCL2, CXCL8, and CXCL10 as significant predictors of poor outcomes. A combined biomarker panel comprising CXCL8, MMP-2, and CCL2 demonstrated excellent predictive accu- racy (AUC ~0.90), with sensitivity ranging from 89% to 98% and specificity ranging from 80% to 90%, depending on the biomarker combination and the selected ROC cut-off.

**Discussion:**

These findings validate key immune biomarkers and support a multi- marker host biomarker panel for early risk stratification and personalized TB management.

## Introduction

Tuberculosis (TB), caused by *Mycobacterium tuberculosis*, remains a leading cause of morbidity and mortality worldwide, particularly in low- and middle-income countries (1). Pulmonary TB (PTB), the most common form, drives most TB transmission and disease burden. Common symptoms include persistent cough, sputum production, haemoptysis, chest pain, fever, night sweats, fatigue, loss of appetite, and weight loss. However, nonspecific or mild symptoms may contribute to delayed diagnosis and continued transmission. Despite effective antimicrobial therapy, TB control is hindered by delayed diagnosis, variable disease progression, and inconsistent treatment responses (2, 3).

The diagnosis of PTB involves clinical assessment combined with bacteriological, molecular, and radiological investigations. Conventional diagnostic methods include sputum smear microscopy, mycobacterial culture, and drug-susceptibility testing. Although culture is highly sensitive, its prolonged turnaround time may delay clinical decision-making. (4, 5). Chest radiography helps identify pulmonary abnormalities, including infiltrates and cavitation, but lacks sufficient specificity to confirm active TB. (6) Recent advances in rapid molecular assays, nucleic acid amplification tests, digital chest radiography, and computer-aided detection have improved the early detection of *M. tuberculosis* and drug resistance. More recently, host biomarker-based approaches are also being explored to improve diagnosis and risk stratification (7).

Effective treatment of PTB requires combination antimicrobial therapy. Drug-susceptible TB is generally treated with an intensive phase, followed by a continuation phase treatment. Recent guidelines have introduced shorter regimens for selected patients, while treatment for drug-resistant TB requires more complex, individualized or standardized regimens. Although standard treatments are generally effective, 10.6% combined unfavourable outcomes such as treatment failure, relapse, or death, underscoring the need for improved prognostic tools and personalized treatment approaches (8, 9).

In recent years, host immune biomarkers have emerged as promising tools for predicting disease severity and treatment response in TB. Cytokines plays an important roles in immune activation and regulation. IL-2 and IFN-γ contribute to T-cell activation and macrophage-mediated immunity, while TNF-α is essential for granuloma formation but may also contribute to inflammation and tissue damage. IL-6 is associated with systemic inflammation and has been identified as a baseline predictor of unfavourable TB treatment outcomes. Other cytokines measured in this study, including IL-1β, IL-10, IL-17A, and GM-CSF, contribute to inflammatory regulation and host defence. Elevated baseline levels of IL-2, IFN-γ, TNF-α, and IL-6 have been associated with disease severity and an increased risk of unfavourable treatment outcomes (10–12).

Chemokines regulate the recruitment and trafficking of immune cells to sites of *M. tuberculosis* infection and contribute to granuloma organization and pulmonary immunopathology. CCL2 promotes monocyte recruitment and has emerged as a strong predictor of unfavourable treatment outcomes. CXCL8 is involved in neutrophil recruitment and may reflect increased inflammation and tissue injury, whereas CXCL10 promotes the recruitment of activated T cells. CCL3, CCL4, and CXCL1 also contribute to the migration and activation of immune cells. Elevated baseline concentrations of CCL2, CXCL8, and CXCL10 have been associated with increased disease severity and unfavourable treatment outcomes, supporting their potential utility as prognostic biomarkers (13–15).

Matrix metalloproteinases (MMPs) are zinc-dependent enzymes involved in extracellular matrix degradation and tissue remodelling. In pulmonary TB, dysregulated MMP activity contributes to lung tissue damage, cavitation, and disease progression. MMP-1, MMP-2, MMP-7, and MMP-9 have been associated with pulmonary tissue remodelling, disease severity, and unfavourable treatment outcomes, suggesting their potential utility as prognostic biomarkers (16–19). Together, cytokines, chemokines, and MMPs reflect complementary aspects of host immune activation, inflammatory-cell recruitment, and pulmonary tissue damage. Our previous studies have identified panels of promising immune mediators including cytokines (10, 11), chemokines (13), and matrix metalloproteinases (16) that are associated with unfavourable TB treatment outcomes. However, many of these biomarkers have been identified in discovery cohorts and require robust validation across populations and clinical settings before they can be translated into clinical practice.

Therefore, we hypothesized that baseline plasma concentrations of selected cytokines, chemokines, and MMPs are associated with subsequent unfavourable TB treatment outcomes and that combining biomarkers from different immune pathways would improve predictive performance. In this study we aim to validate these biomarkers using well-characterized samples from four independent cohorts within the RePORT (Regional Prospective Observational Research for Tuberculosis) India consortium, which provides longitudinal data and standardized protocols for robust cross-cohort analysis. By assessing the reproducibility and predictive performance of cytokines, chemokines, and MMPs, we seek to identify a refined set of biomarkers consistently associated with unfavourable outcomes. Importantly, emerging evidence suggests that these biomarkers demonstrate good reproducibility when evaluated in independent cohorts, showing consistent trends comparable to those observed in initial discovery studies (7, 20). In particular, cytokines such as IL-2, IFN-γ and IL-6 chemokines like CCL-2, CXCL-8 and CXCL-10, and MMPs including MMP-1, MMP-2, MMP-7, MMP-9 have shown stable expression patterns and significant associations with unfavourable treatment outcomes across different study populations (7, 15, 17, 18). This reproducibility strengthens their potential utility as reliable immune biomarkers for prognostication in TB.

Overall, this study aims to refine a robust panel of immune biomarkers for predicting TB treatment outcomes. Such validation is a critical step toward translating biomarker discoveries into clinically applicable tools. Ultimately, identifying a reliable panel of immune biomarkers may facilitate early identification of high-risk individuals, inform personalized treatment strategies, and improve overall TB management and outcomes.

## Materials and methods

### Ethics statement

The validation study cohort was approved by the ICMR-NIRT Internal Ethical Committee (Ethics number: 150/NIRT-IEC/2020) and Prof. M. Viswanathan Diabetes Research Centre Ethics Committees. Informed written consent was obtained from all participants. All the methods were performed in accordance with the relevant institutional ethical committee guidelines.

### Study design

Participants were enrolled from the RePORT India consortium clinical study cohort across four distinct sites. Inclusion criteria were newly diagnosed pulmonary TB patients, aged 18 to 67 years with clinical or microbiological confirmation. Exclusion criteria were treatment for any prior episode of TB disease, more than 7 days treatment for incident TB, more than 7 doses of a fluoroquinolone within 30 days of screening, drug-resistant *Mycobacterium tuberculosis* isolate from the enrollment sputum culture, pregnant or nursing, and HIV-seropositive or taking immunosuppressive drugs, diagnosed autoimmune or autoinflammatory diseases, active co-infections or other clinically significant infectious diseases, and current or previous malignancy. The diagnosis of PTB was established by positive sputum culture on solid media (Lowenstein-Jensen media) with compatible chest X ray. TB treatment was managed by government clinics in Chennai according to the Revised National Tuberculosis Control Program standards. Participants were followed monthly through the course of the 6-month treatment and at 3-month intervals thereafter until 1 year after completion of treatment. Our study is case-control study with cases defined as unfavourable treatment outcomes and matched in a 1:2 ratio to controls who were defined as having a recurrence-free cure until the end of study. Cure was defined as negative sputum cultures at months 5 and 6 of treatment without recurrent disease during follow-up. Treatment failure was defined as positive sputum culture at month 5 or 6. Death comprised all-cause mortality during TB treatment. Recurrence was defined as initial cure at month 6 but culture proven occurrence of disease before the end of the study. There were 40 treatment failures, 17 deaths, and 17 recurrences in the Case cohort. No formal sample size calculation was performed for this study because the analysis utilized archived samples obtained from the RePORT India Consortium Central Biorepository, and all eligible available samples meeting the study criteria were included. In our previously published study, the discovery cohort comprised 68 cases and 133 controls. To ensure adequate statistical power for biomarker validation and to strengthen the robustness of the findings, we aimed to include at least the same number of participants as the discovery cohort. Accordingly, the validation cohort consisted of 74 cases and 161 controls. Peripheral blood was collected in heparin tubes. Following centrifugation, plasma was collected and stored at −80°C until further analysis. Sample collection was performed at baseline (before treatment initiation).

### Multiplex ELISA bead-based immunoassay

A multiplex enzyme-linked immunosorbent assay (ELISA) for cytokines, chemokines and matrix metalloproteinases was conducted to examine antigen-specific in unfavourable treatment outcomes (cases, n =74) and favourable treatment outcomes (controls, n =161). The assay followed the manufacturer’s instructions using the Human Magnetic Luminex Discovery Assay (R&D Systems). Stimulated plasma cytokine, chemokines and matrix metalloproteinases levels were measured using Luminex 200 with xMAP technology (Bio-Rad, Hercules, CA). The lowest detection limits for cytokines, chemokines and matrix metalloproteinases were as follows:IL-2, 3.9 pg/mL; IL-6, 2.2 pg/mL; IL-10, 3.6 pg/mL; IL-17A, 2.5 pg/mL; IFN-γ, 2.3 pg/mL; TNF-α, 3.0 pg/mL; GM-CSF, 3.4 pg/mL; CCL2/MCP-1, 1.8 pg/mL; CCL3/MIP-1α, 2.9 pg/mL; CCL4/MIP-1β, 1.7 pg/mL; CXCL1/GROα, 1.9 pg/mL; IL-8/CXCL8, 3.2 pg/mL; CXCL10/IP-10, 3.3 pg/mL; MMP-1, 5 pg/mL; MMP-2, 20 pg/mL; MMP-7, 3 pg/mL; and MMP-9, 5 pg/mL. which were analyzed between cases and cured controls.

### Statistical analysis

Statistical analyses were performed to evaluate differences in cytokine, chemokine, and matrix metalloproteinase (MMP) concentrations between cases and controls, and to assess the diagnostic performance of candidate biomarkers measured using the Human Magnetic Luminex Discovery Assay (LXSAHM 18-plex, R&D Systems). Data were analyzed using GraphPad Prism (version 11) and R statistical software. Continuous variables were summarized as median and interquartile range (IQR) due to non-normal distribution. Group comparisons were performed using the Mann–Whitney U test, with a two-tailed *p* value <0.05 considered statistically significant.

Receiver Operating Characteristic (ROC) curve analysis was used to evaluate the diagnostic performance of individual biomarkers. The area under the curve (AUC) was calculated, and optimal cut-off values were determined using the Youden index. Sensitivity, specificity, positive predictive value (PPV), negative predictive value (NPV), and overall accuracy were calculated with 95% confidence intervals (CI). Associations between biomarkers and disease status were assessed using univariable and multivariable logistic regression models. Biomarker concentrations were log-transformed prior to analysis, and adjusted models included age, gender, body mass index, smoking status, and alcohol consumption. Odds ratios (OR) and adjusted odds ratios (aOR) with 95% CI were reported.

Feature selection was performed using a Random Forest algorithm, with variable importance assessed based on mean decrease in accuracy. Principal Component Analysis (PCA) was applied for dimensionality reduction and visualization of clustering patterns. Stability selection analysis was conducted using bootstrap resampling to estimate the consistency of biomarker selection. Finally, multivariable logistic regression models were used to evaluate the combined diagnostic performance of selected biomarkers, and predicted probabilities were used to generate ROC curves for biomarker combinations.

## Result

### Demographic profile of the study population

The median age was 42 years for cases and 39 years for controls. As tuberculosis is predominantly observed in males, 60 males were included in the case group and 125 males in the control group. There were no significant differences between the groups in terms of age, gender, BMI, cough status, smoking, alcohol use, chest X-ray score, and the presence of cavities. However, significant differences were observed in the diabetes group (P = 0.014), smear status (P = 0.0045), and solid culture status (P = 0.0012). In contrast, no significant difference was observed in liquid culture status (**Table 1**).

**Table 1:** Demographic and clinical characteristic of the cohort.

Characteristic	All (n=235)	Case (n=74)	Control (n=161)	P Value
Age, median (Range)	39 (18-67)	42 (18-67)	39 (18-67)	
Sex				
Male	185	60	125	0.5494
Female	50	14	36	
BMI, median (Range)	17.83 (11-34.2)	16.8 (11-27.6)	17.8 (13.2-34.2)	
Cough				
Yes	234	74	160	0.4969
No	1	0	1	
Cough Dur, mean(range)	14.7 (1-90)	22.7 (2-90)	14.7 (1-90)	
DM status (HbA1c), mean(range)	6.8 (4.1-14.5)	6.5 (4.1-14.5)	6.8 (4.2-13.7)	
DM Group				
Non-Diabetic	33	14	19	0.014
Pre-Diabetic	26	14	12	
Diabetic	45	10	35	
Unknown	131	36	95	
Smoking				
Current	49	15	34	0.7372
Former	35	13	22	
Never	151	46	105	
Alcohol				
Yes	132	46	86	0.4272
No	98	27	71	
Unknown	5	1	4	
Cavity				
Yes	153	44	109	0.4685
No	71	26	45	
Unknown	11	4	7	
Smear				
Positive	114	46	68	0.0045
Negative	121	28	93	
Solid Culture				
Positive	120	48	72	0.0012
Negative	54	19	35	
Contaminated	4	0	4	
Not done	57	7	50	
Chest X-Ray Score, mean(range)	58 (0-120)	62.8 (0-120)	58 (0-120)	
Liquid culture				
Positive	166	49	117	0.7235
Negative	34	13	21	
Contaminated	4	1	3	
Not done	31	11	20	

### Plasma cytokine levels as an immune markers for unfavourable treatment outcomes

To determine the association between baseline pre-treatment cytokine levels and unfavourable treatment outcomes, we measured plasma cytokines including IL-2, IFN-γ, TNF-α, IL-1β, IL-17A, IL-10, IL-6, and GM-CSF in both the case and control groups. Among these, IL-2, IFN-γ, TNF-α, and IL-6 showed strong statistical significance (p < 0.0001), while IL-1β also demonstrated a significant difference (p = 0.0090) and there is no significance was observed in IL-17a, IL-10 and GM CSF levels between the cohorts (**Figure 1A**). To further distinguish plasma cytokines levels between cases and controls, Receiver Operating Characteristic (ROC) analysis was performed for the significantly associated cytokines. IL-2 demonstrated an AUC of 0.8 with a sensitivity of 81% and specificity of 81%. IFN-γ showed an AUC of 0.69 with sensitivity of 82% and specificity of 61%. IL-6 exhibited an AUC of 0.7 with sensitivity of 50% and specificity of 81%. TNF-α showed an AUC of 0.8 with sensitivity of 76% and specificity of 87%. IL-1β showed an AUC of 0.6 with sensitivity of 61% and specificity of 61% (**Figure 1B**). Based on the p-values and confidence intervals, three cytokines were identified as potential biomarkers for predicting patients at higher risk of unfavourable treatment outcomes. Accordingly, a combined ROC analysis was performed using IL-2, IFN-γ, and IL-6, representing distinct immune pathways and capturing multiple aspects of the host immune response. ROC analysis of different cytokine combinations like IL-2+IFN-γ, IL-2+IL-6 and IL-2+IFN-γ+IL-6 produced an AUC of 0.9 discrimination with sensitivities of 80%, 84% and 81% and Specificities of 90%, 87% and 90% respectively. The combination of IFN-γ+IL-6 produced an AUC of 0.8 with sensitivity of 78% and specificity of 79% (**Figure 1C**). These findings suggest that a combination of immune markers provides better predictive value for identifying unfavourable treatment outcomes.

**Figure 1:**
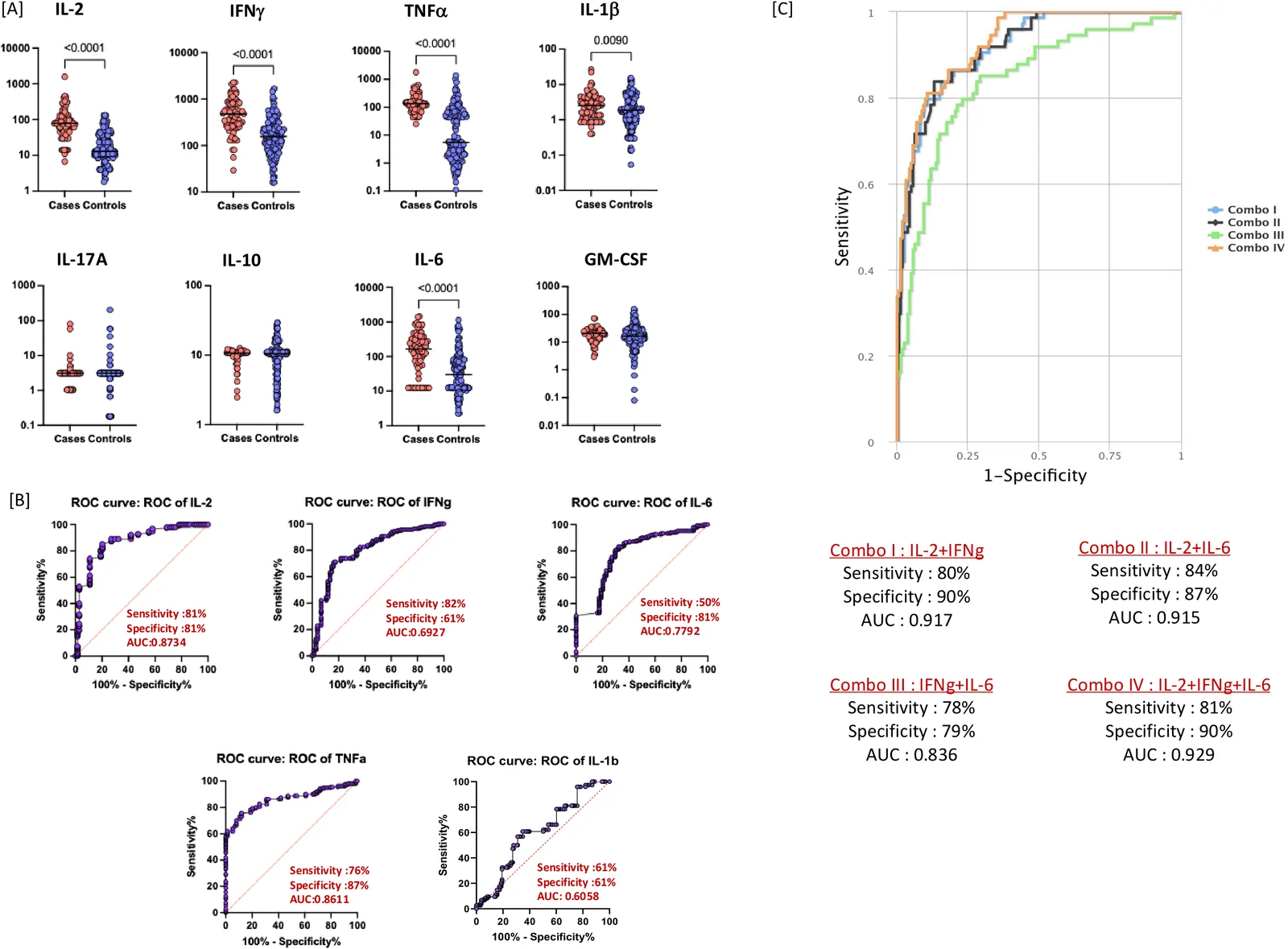
Comparison and diagnostic performance of cytokines. **(A)** Distribution of cytokine concentrations (IL-2, IFN-γ, TNF-α, IL-1β, IL-17A, IL-10, IL-6, and GM-CSF) in cases and controls. Data are presented as scatter plots with median indicated. Group comparisons were performed using the Mann–Whitney U test, and corresponding p-values are shown above each plot. **(B)** Receiver operating characteristic (ROC) curves illustrating the diagnostic performance of selected individual cytokines (IL-2, IFN-γ, TNF-α, IL-1β). **(C)** ROC curves of combined cytokine panels evaluating multi-marker diagnostic performance.

### Plasma chemokine levels as an immune markers for unfavourable treatment outcomes

To evaluate the association of chemokines, plasma levels of CCL2, CCL3, CCL4, CXCL1, CXCL8, and CXCL10 were measured. Among these, CCL2, CCL4, CXCL1, CXCL8, and CXCL10 showed statistically significant differences with a p-value of <0.0001, whereas CCL3 did not show significant association (**Figure 2A**). To further distinguish plasma chemokine levels between cases and controls, Receiver Operating Characteristic (ROC) analysis was performed for the significantly associated chemokines. CXCL8 demonstrated an AUC of 0.9 with a sensitivity of 91% and specificity of 62%. CXCL10 showed an AUC of 0.79 with sensitivity of 74% and specificity of 71%. CCL2 exhibited an AUC of 0.89 with sensitivity of 80% and specificity of 80%, while CCL4 showed an AUC of 0.8 with sensitivity of 70% and specificity of 71% (**Figure 2B**). Further, combinational ROC analyses were performed using CCL2, CXCL8, and CXCL10 to determine whether combining chemokines improves diagnostic performance. The combinations CCL2+CXCL8, CCL2+CXCL10, CXCL8+CXCL10 and CCL2+CXCL8+CXCL10 produced an AUC of 0.9 excellent discrimination with sensitivities of 97%, 89%, 83% and 91% and specificities of 85%, 85%, 89% and 93%, respectively. (**Figure 2C**).

**Figure 2:**
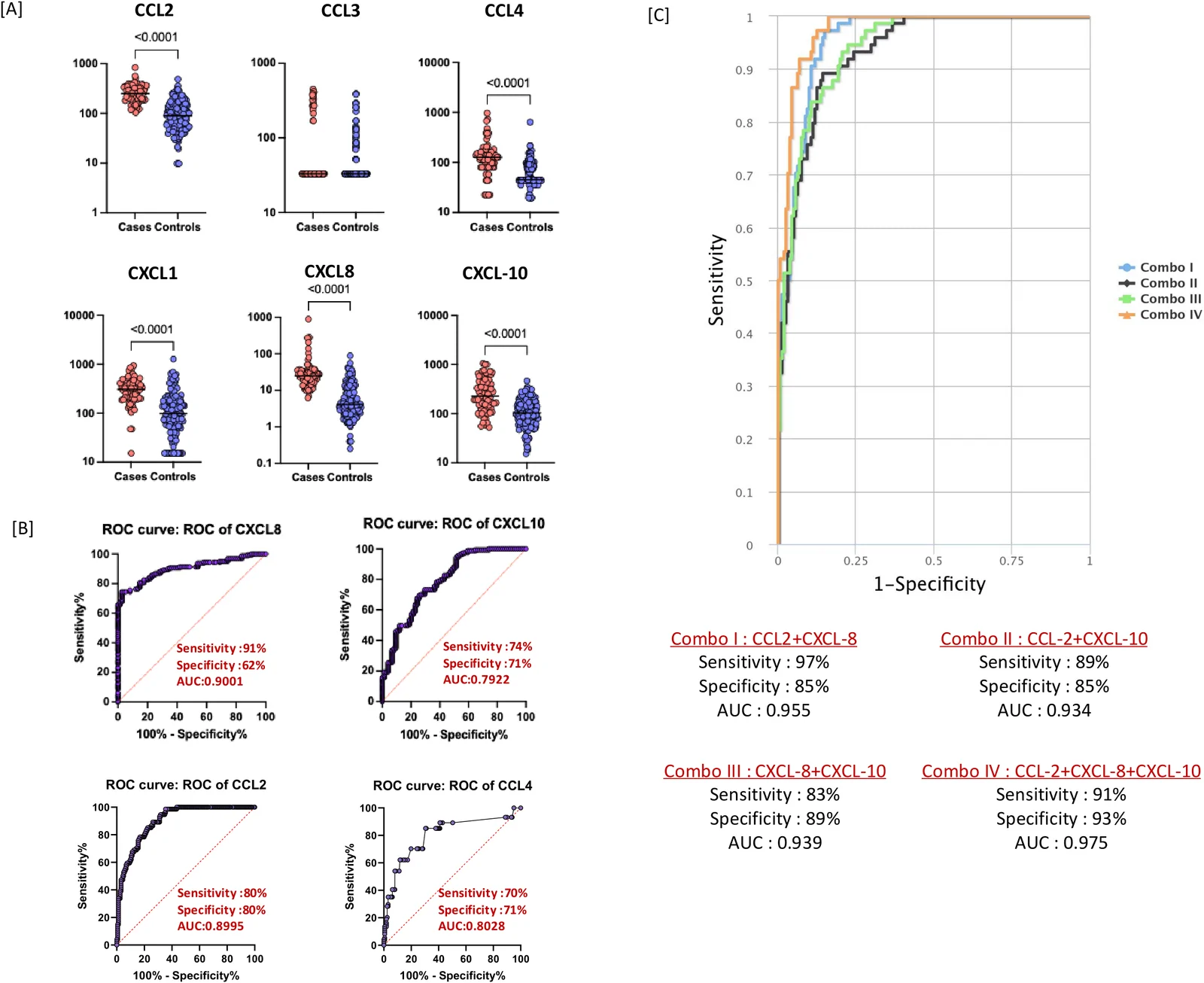
Comparison and diagnostic performance of chemokines. **(A)** Distribution of chemokine concentrations (CCL2, CCL3, CCL4, CXCL1, CXCL8, and CXCL10) in cases and controls. Data are presented as scatter plots with median indicated. Group comparisons were performed using the Mann–Whitney U test, and corresponding p-values are shown above each plot. **(B)** Receiver operating characteristic (ROC) curves illustrating the diagnostic performance of selected individual chemokines (CXCL8, CXCL10, CCL2, and CCL4). **(C)** ROC curves of combined chemokine panels evaluating multi-marker diagnostic performance. Combinations include CCL2 + CXCL-8 (Combo I), CCL2 + CXCL-10 (Combo II), CXCL-8 + CXCL-10 (Combo III), and CCL2 + CXCL-8 + CXCL-10 (Combo IV). Combo IV (CCL2 + CXCL8 + CXCL10) achieved the highest diagnostic performance with an AUC of 0.929, a sensitivity of 90%, and a specificity of 82%. The combination of markers significantly improves diagnostic power compared to any single chemokine used in isolation.

### Plasma MMPs levels as an immune markers for unfavourable treatment outcomes

Similar to the above mentioning parameters we have also measure the plasma levels of matrix metalloproteinases (MMPs) such as MMP-1, MMP-2, MMP-7 and MMP-9 in both cohorts. Among which MMP-2, MMP-7 and MMP-9 showed strong statistically significant between the cohorts with the P value of < 0.0001. and MMP-1 showed significant result with the P value of 0.0079 (**Figure 3A**). As shown in the **Figure 3B**, ROC analysis was performed on MMP-1(AUC = 0.6), MMP-2 (AUC = 0.8), MMP-7 (AUC = 0.7) and MMP-9 (AUC = 0.8) with sensitivity 66%, 89%, 78% and 90% and specificity of 44%, 70%, 64% and 63% respectively. Among which MMP-2 showed the more balanced significance with AUC of 0.8. In addition to that combined ROC analysis was performed using different type of combination of MMPs like MMP-1+MMP-2(AUC = 0.8), MMP-1+MMP-7(AUC = 0.7), MMP-1+MMP-9(AUC = 0.8), MMP-2+MMP-7(AUC = 0.8), MMP-2+MMP-9(AUC = 0.8), MMP-7+MMP-9(AUC = 0.8), MMP-1+MMP-2+MMP-7(AUC = 0.8), MMP-1+MMP-2+MMP-9(AUC = 0.8), MMP-1+MMP-7+MMP-9(AUC = 0.8), MMP-2+MMP-7+MMP-9(AUC = 0.9), MMP-1+MMP-2+MMP-7+MMP-9(AUC = 0.9) to predict which group of MMPs produce more promising results. At the end AUC rages from (0.7 to 0.9) with sensitivity ranges from 73% to 91% (74%, 87%, 73%, 85%, 78%, 75%, 91%, 81%, 75%, 82% and 85%) and specificity ranges from 60 to 89%(85%, 60%, 83%, 74%, 88%, 87%, 67%, 89%, 87%, 86% and 84%). Overall MMP-2+MMP-7+MMP-9 and all four MMPs together produce AUC of 0.9 (**Figure 3C**).

**Figure 3:**
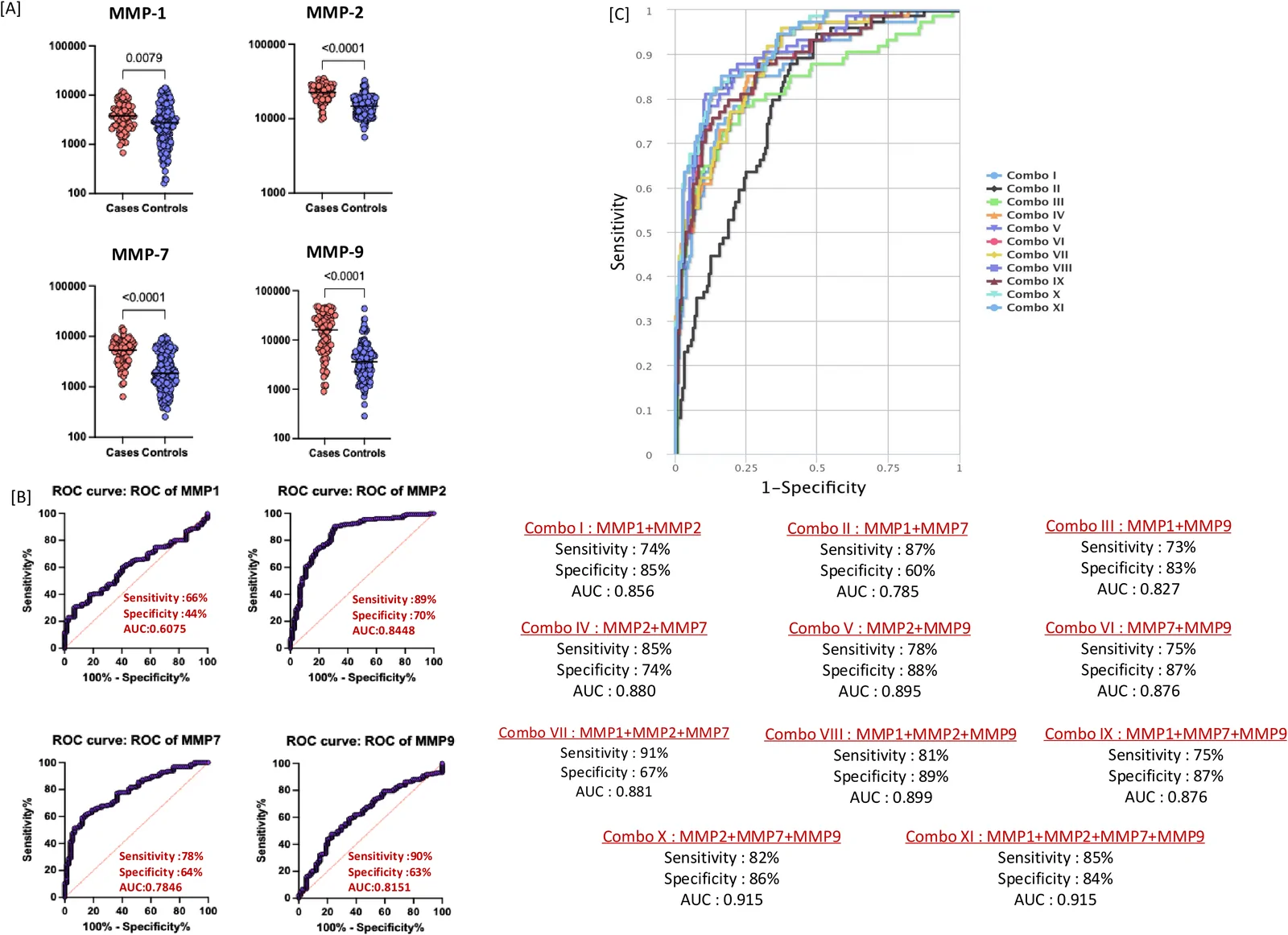
Comparison and diagnostic performance of matrix metalloproteinases (MMPs). **(A)** Distribution of MMP concentrations (MMP-1, MMP-2, MMP-7, and MMP-9) in cases and controls. Data are presented as scatter plots with median indicated. Group comparisons were performed using the Mann–Whitney U test, and corresponding p-values are shown above each plot. **(B)** Receiver operating characteristic (ROC) curves illustrating the diagnostic performance of selected individual MMPs (MMP-1, MMP-2, MMP-7, and MMP-9). **(C)** ROC curves of combined MMP panels evaluating multi-marker diagnostic performance.

### Diagnostic performance of biomarkers

The diagnostic performance of individual cytokines, chemokines, and matrix metalloproteinases (MMPs) for distinguishing unfavourable treatment outcomes from favourable outcomes is summarized in **Table 2**. Among the cytokines, IL-2 demonstrated the highest discriminatory ability with an AUC of 0.8, along with sensitivity (72%) and specificity (90%) with 80% of positive predictive value, followed by TNF-α, IFN-γ, IL-6(AUC = 0.7), IL-10, IL-1β(AUC = 0.6), IL-17A(AUC = 0.5), GMCSF(AUC = 59%) with 63%, 57%, 64%, 36%, 45%, 46%, 35% sensitivity and 93%, 91%, 86%, 97%, 78%, 71%, 84% specificity respectively. Among chemokines, CXCL-8 demonstrated AUC of 0.8 with 64% sensitivity and 98% specificity with 97% of positive predictive value followed by CCL-2(AUC = 78%), CXCL-1(AUC = 76%), CCL-4(AUC = 74%), CCL-3(71%), CXCL-10(AUC = 69%) showed 56%, 60%, 56%, 68%, 53% sensitivity and 99%, 91%, 91%, 73%, 86% specificity. For MMPs, MMP-2 demonstrated the strongest diagnostic performance with an AUC of 0.82, 77% sensitivity and 86% specificity with 67% positive predictive value, followed by MMP-9(AUC = 0.8), MMP-7(AUC = 0.7) and MMP-1(AUC = 64%) with 74%, 50%, 38% sensitivity and 85%, 91%, 91% specificity respectively Among the evaluated analytes, IL-2, CXCL-8, CCL-2, and MMP-2 showed comparatively better diagnostic performance than the others, demonstrating relatively consistent and balanced accuracy. (**Table 2**).

**Table 2:** Diagnostic performance of selected immunological biomarkers for disease detection.

Biomarker	TP	FP	FN	TN	Cut-off	Sensitivity	Specificity	PPV	NPV	Accuracy	ROC Area
IL1b	45	56	29	105	2.12	**44.6** (34.7, 54.8)	**78.4** (70.4, 85.0)	**60.8** (48.8, 72.0)	**65.2** (57.3, 72.5)	**63.8** (57.3, 70.0)	**61.5** (55.5, 67.5)
IL2	59	23	15	138	46.00	**72.0** (60.9, 81.3)	**90.2** (84.3, 94.4)	**79.7** (68.8, 88.2)	**85.7** (79.3, 90.7)	**83.8** (78.5, 88.3)	**81.1** (75.6, 86.5)
IL6	52	29	22	132	86.00	**64.2** (52.8, 74.6)	**85.7** (79.2, 90.8)	**70.3** (58.5, 80.3)	**82.0** (75.2, 87.6)	**78.3** (72.5, 83.4)	**75.0** (69.0, 80.9)
IL10	73	130	1	31	12.14	**36.0** (29.4, 43.0)	**96.9** (83.8, 99.9)	**98.6** (92.7, 100.0)	**19.3** (13.5, 26.2)	**44.3** (37.8, 50.9)	**66.4** (61.9, 70.9)
IL17A	16	19	58	142	1.04	**45.7** (28.8, 63.4)	**71.0** (64.2, 77.2)	**21.6** (12.9, 32.7)	**88.2** (82.2, 92.7)	**67.2** (60.8, 73.2)	**58.4** (49.4, 67.3)
IFNg	62	47	12	114	233.00	**56.9** (47.0, 66.3)	**90.5** (84.0, 95.0)	**83.8** (73.4, 91.3)	**70.8** (63.1, 77.7)	**74.9** (68.8, 80.3)	**73.7** (68.3, 79.0)
TNFa	65	39	9	122	67.94	**62.5** (52.5, 71.8)	**93.1** (87.4, 96.8)	**87.8** (78.2, 94.3)	**75.8** (68.4, 82.2)	**79.6** (73.8, 84.5)	**77.8** (72.7, 83.0)
GMCSF	69	131	5	27	32.26	**34.5** (27.9, 41.5)	**84.4** (67.2, 94.7)	**93.2** (84.9, 97.8)	**17.1** (11.6, 23.9)	**41.4** (35.0, 48.0)	**59.4** (52.2, 66.6)
CCL2	73	57	1	104	119.18	**56.2** (47.2, 64.8)	**99.0** (94.8, 100.0)	**98.6** (92.7, 100.0)	**64.6** (56.7, 72.0)	**75.3** (69.3, 80.7)	**77.6** (73.2, 82.0)
CCL3	19	9	55	152	169.54	**67.9** (47.6, 84.1)	**73.4** (66.9, 79.3)	**25.7** (16.2, 37.2)	**94.4** (89.7, 97.4)	**72.8** (66.6, 78.4)	**70.6** (61.3, 80.0)
CCL4	63	49	11	112	79.79	**56.2** (46.6, 65.6)	**91.1** (84.6, 95.5)	**85.1** (75.0, 92.3)	**69.6** (61.8, 76.6)	**74.5** (68.4, 79.9)	**73.7** (68.4, 78.9)
CXCL1	62	41	12	120	178.94	**60.2** (50.1, 69.7)	**90.9** (84.7, 95.2)	**83.8** (73.4, 91.3)	**74.5** (67.1, 81.1)	**77.4** (71.6, 82.6)	**75.6** (70.2, 80.9)
CXCL8	72	41	2	119	9.78	**63.7** (54.1, 72.6)	**98.3** (94.2, 99.8)	**97.3** (90.6, 99.7)	**74.4** (66.9, 80.9)	**81.6** (76.1, 86.4)	**81.0** (76.4, 85.6)
CXCL10	55	49	19	112	146.50	**52.9** (42.8, 62.8)	**85.5** (78.3, 91.0)	**74.3** (62.8, 83.8)	**69.6** (61.8, 76.6)	**71.1** (64.8, 76.8)	**69.2** (63.5, 74.9)
MMP1	69	112	5	49	1400	**38.1** (31.0, 45.6)	**90.7** (79.7, 96.9)	**93.2** (84.9, 97.8)	**30.4** (23.4, 38.2)	**50.2** (43.6, 56.8)	**64.4** (59.2, 69.7)
MMP2	51	15	23	145	20000	**77.3** (65.3, 86.7)	**86.3** (80.2, 91.1)	**68.9** (57.1, 79.2)	**90.6** (85.0, 94.7)	**83.8** (78.4, 88.2)	**81.8** (76.1, 87.5)
MMP7	65	65	9	94	2300	**50.0** (41.1, 58.9)	**91.3** (84.1, 95.9)	**87.8** (78.2, 94.3)	**59.1** (51.1, 66.8)	**68.2** (61.8, 74.2)	**70.6** (65.5, 75.7)
MMP9	48	17	26	144	9000	**73.8** (61.5, 84.0)	**84.7** (78.4, 89.8)	**64.9** (52.9, 75.6)	**89.4** (83.6, 93.7)	**81.7** (76.2, 86.4)	**79.3** (73.2, 85.3)

Cut-off values were derived using the Youden index.

Sensitivity, specificity, predictive values (PPV, NPV), accuracy, and ROC AUC are reported with 95% confidence intervals.

TP, True Positive; FP, False Positive; FN, False Negative; TN, True Negative.

The table presents the diagnostic accuracy of 18 biomarkers, including cytokines and matrix metalloproteinases. Performance metrics include true positive (TP), false positive (FP), false negative (FN), and true negative (TN) counts, along with optimal cut-off values determined via the Youden index. Sensitivity, specificity, predictive values (PPV, NPV), overall accuracy, and Area Under the Receiver Operating Characteristic Curve (ROC AUC) are provided with 95% confidence intervals (CI).

The sensitivity and specificity of all measured parameters are highlighted in bold.

### Association of biomarkers with disease status

The association between baseline levels of inflammatory biomarkers and unfavourable TB treatment outcomes was assessed using logistic regression analysis (**Table 3**). In the unadjusted analysis, several cytokines, chemokines, and MMPs showed significant associations with disease status. Among cytokines, IL-2 (OR: 5.6), IL-6 (OR: 2.16), IFN-γ (OR: 3.76), TNF-α (OR: 2.91) showed significant association with the P value of <0.001. IL-1β also showed a modest but significant association (OR: 1.48, p=0.010), whereas IL-10, IL-17A, and GM-CSF were not significantly associated. Among chemokines, CCL2 demonstrated the strongest association with unfavourable outcomes (OR: 29.2, p<0.001), followed by CXCL8 (OR: 7.16), CCL4 (OR: 6.41), CXCL10 (OR: 5.82) and CXCL1 (OR: 4.45) all with highly significant p-values (<0.001). CCL3 also showed a significant but comparatively weaker association (OR: 1.75, p=0.002). For MMPs, all markers were significantly associated with disease status, with MMP-1 (OR: 1.63, p=0.004), MMP-2 (OR: 1.03, p<0.001), MMP-7 (OR: 1.02, p<0.001), and MMP-9 (OR: 1.04, p<0.001) showing consistent positive associations.

**Table 3:** Association of inflammatory cytokines, chemokines, and MMPs with disease status.

Variable	Unadjusted			Adjusted		
	OR	95% CI	p-value	aOR	95% CI	p-value
IL1b	1.48	[1.10, 2.00]	0.010	1.52	[1.10, 2.09]	0.010
IL2	5.60	[3.57, 8.78]	<0.001	5.38	[3.40, 8.51]	<0.001
IL6	2.16	[1.70, 2.74]	<0.001	2.16	[1.68, 2.78]	<0.001
IL10	1.59	[0.91, 2.78]	0.102	1.61	[0.90, 2.86]	0.106
IL17A	1.12	[0.83, 1.51]	0.460	1.28	[0.92, 1.78]	0.149
IFNg	3.76	[2.52, 5.60]	<0.001	3.70	[2.45, 5.60]	<0.001
TNFa	2.91	[2.08, 4.06]	<0.001	3.15	[2.20, 4.51]	<0.001
GMCSF	1.25	[0.90, 1.75]	0.187	1.28	[0.88, 1.85]	0.190
CCL2	29.20	[11.71, 72.81]	<0.001	38.00	[13.75, 104.98]	<0.001
CCL3	1.75	[1.22, 2.51]	0.002	1.86	[1.27, 2.74]	0.002
CCL4	6.41	[3.60, 11.41]	<0.001	6.41	[3.50, 11.72]	<0.001
CXCL1	4.45	[2.83, 6.98]	<0.001	5.41	[3.22, 9.07]	<0.001
CXCL8	7.16	[4.22, 12.15]	<0.001	8.26	[4.57, 14.92]	<0.001
CXCL10	5.82	[3.46, 9.79]	<0.001	6.92	[3.81, 12.56]	<0.001
MMP1	1.63	[1.17, 2.27]	0.004	1.48	[1.02, 2.14]	0.040
MMP2	1.03	[1.01, 1.05]	<0.001	1.03	[1.01, 1.05]	<0.001
MMP7	1.02	[1.01, 1.04]	<0.001	1.02	[1.01, 1.04]	<0.001
MMP9	1.04	[1.01, 1.03]	<0.001	1.04	[1.01, 1.03]	<0.001

OR, Odds Ratio; aOR, adjusted Odds Ratio; CI, Confidence Interval.

Adjusted model was adjusted for age, gender, body mass index, smoking and alcohol.

Biomarker observations were transformed using log before performing the model.

Results from logistic regression analyses are presented as crude odds ratios (OR) and adjusted odds ratios (aOR) with corresponding 95% confidence intervals (CI). The adjusted model accounts for potential confounders including age, gender, body mass index, smoking, and alcohol consumption. All biomarker concentrations were log-transformed prior to modelling to normalize distribution. Values with p < 0.05 indicate statistically significant associations with the disease state.

After adjustment for potential confounders (age, gender, BMI, smoking, and alcohol use), these associations remained largely unchanged. IL-2 (aOR: 5.38), IFN-γ (aOR: 3.70), TNF-α (aOR: 3.15), and IL-6 (aOR: 2.16) continued to show strong independent associations with the P value of <0.001. Notably, CCL2 remained the most robust predictor (aOR: 38.00, p<0.001), followed by CXCL8 (aOR: 8.26), CXCL10 (aOR: 6.92), and CCL4 (aOR: 6.41). Among matrix metalloproteinases, MMP-2, MMP-7, MMP-9 (aOR:1, p<0.001) and MMP-1 (aOR:1.48, p=0.040) retained statistical significance in the adjusted model, further supporting their role as independent predictors of unfavourable TB treatment outcomes.

### Random forest variable importance and principal component analysis

Biomarker Importance Ranking: Biomarkers were ranked based on their Mean Decrease in Accuracy. The selection of the final panel (top-selected features) resulted in a notable performance refinement, with classification accuracy shifting from Accuracy of all the Biomarkers in the full model to the Accuracy of the Selected Biomarkers in the optimized model (**Figure 4A**). Higher importance values indicate a greater contribution to the predictive performance of the model. PCA Biplot of Selected Biomarkers: Principal Component Analysis visualization of the optimized feature set. Individual points represent patients, color-coded by clinical group, with 95% confidence ellipses overlaid. Red vectors (arrows) indicate the loading of each biomarker on PC1 and PC2, illustrating the direction and strength of their influence on group separation and the correlations between selected predictors.

**Figure 4:**
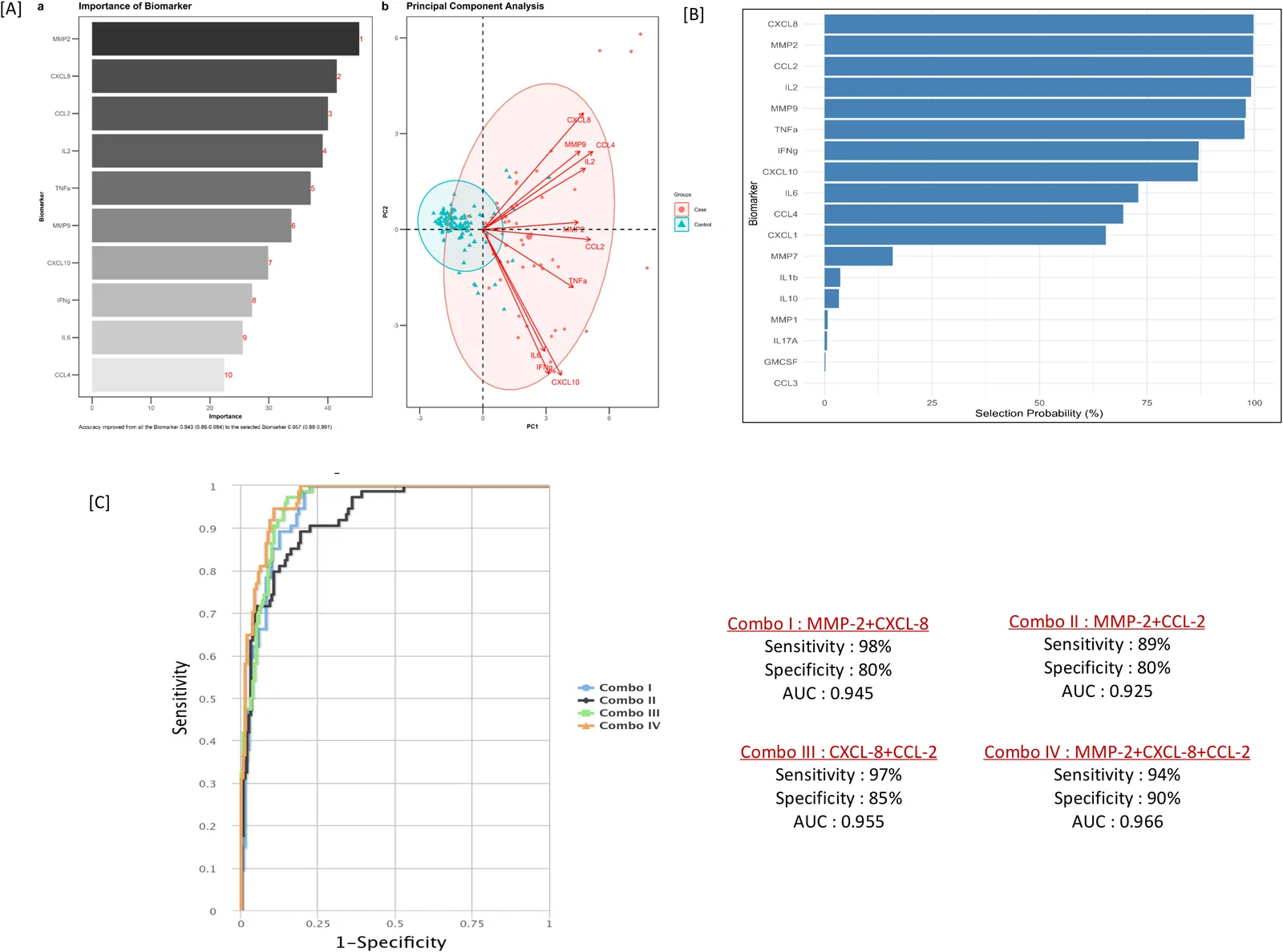
**(A)** Random forest (RF) variable importance and principal component analysis (PCA): (a) Biomarker Importance Ranking: Biomarkers are ranked based on their Mean Decrease in Accuracy. The selection of the final panel (top-selected features) resulted in a notable performance refinement, with classification accuracy shifting from Accuracy of all the Biomarkers in the full model to the Accuracy of the Selected Biomarkers in the optimized model. Higher importance values indicate a greater contribution to the predictive performance of the model. (b) PCA Biplot of Selected Biomarkers: Principal Component Analysis visualization of the optimized feature set. Individual points represent patients, color-coded by clinical group, with 95% confidence ellipses overlaid. Red vectors (arrows) indicate the loading of each biomarker on PC1 and PC2, illustrating the direction and strength of their influence on group separation and the correlations between selected predictors. **(B)** Feature Selection Stability Analysis.To evaluate the robustness of the selected biomarker panel, a bootstrap-based stability analysis was performed (1000 iterations). The plot displays the Selection Probability (%) for each biomarker, representing the frequency with which a specific variable was identified among the top n predictors across all bootstrap resamples. High selection probabilities (approaching 100%) indicate features that are consistently influential despite variations in the dataset, whereas lower probabilities suggest features sensitive to sample composition. This analysis validates the reliability of the identified biomarkers for consistent disease classification. **(C)** ROC curves of optimized cross-family biomarker combinations evaluating multi-marker diagnostic performance.

### Feature selection stability analysis

To evaluate the robustness of the selected biomarker panel, a bootstrap-based stability analysis was performed (1000 iterations). The plot (**Figure 4B**) displays the Selection Probability (%) for each biomarker, representing the frequency with which a specific variable was identified among the top n predictors across all bootstrap resamples. High selection probabilities (approaching 100%) indicate features that are consistently influential despite variations in the dataset, whereas lower probabilities suggest features sensitive to sample composition. This analysis validates the reliability of the identified biomarkers for consistent disease classification.

### Selection of potential biomarker combinations

Based on the preceding analysis, the three most promising biomarkers with potential to predict unfavourable treatment outcomes were CXCL8, MMP-2, and CCL2. To assess their predictive performance, receiver operating characteristic (ROC) analyses were performed using different biomarker combinations (**Figure 4C**). All tested combinations demonstrated high diagnostic accuracy, with an area under the curve (AUC) of 0.9. The sensitivity and specificity ranged from 89–98% and 80–90%, respectively. The combination of MMP-2, CXCL-8, and CCL-2 showed the highest specificity and AUC, establishing it as the lead diagnostic combination. Future work should focus on prioritising a single lead biomarker combination for validation in prospective, multi-centre international cohorts, as a critical step toward subsequent diagnostic clinical trials aimed at treatment stratification. In parallel, efforts to translate this biomarker signature into an affordable and scalable diagnostic platform through appropriate partnerships will be essential for clinical implementation.

## Discussion

TB continues to be one of the major public health problems especially in endemic countries like India. Recurrence TB continues to be a significant concern and the absence of reliable biomarker to predict the treatment further complicates the effective treatment management. In this study, we performed a comprehensive validation of previously identified immune biomarkers across independent cohorts within the RePORT India consortium, demonstrating that cytokines, chemokines, and matrix metalloproteinases (MMPs) are significantly associated with unfavourable TB treatment outcomes. Importantly, several biomarkers including IL-2, IFN-γ, TNF-α, IL-6, CXCL8, CXCL10, CCL2, and MMP-2, MMP-7, and MMP-9 showed consistent reproducibility and discriminatory ability, reinforcing prior discovery-phase findings and highlighting their robustness across diverse patient populations.

Our findings are in agreement with earlier studies showing that elevated pro-inflammatory cytokines such as IL-6 and TNF-α reflect heightened immune activation and disease severity in TB (10, 11). Similarly, IFN-γ and IL-2, which are central to T-cell mediated immunity, have been associated with bacterial burden and treatment response dynamics (12). IL-2 and IFN-γ promote T-cell activation and macrophage-mediated control of *M. tuberculosis*, while TNF-α supports granuloma formation but may also cause inflammation and tissue damage. Elevated IL-6 may indicate persistent immune activation. Thus, increased levels of these cytokines may reflect dysregulated immune responses associated with disease severity and unfavourable treatment outcomes. Significant elevations in TNF-α and IFN-γ levels in TB patients compared to controls were reported highlighting their role in host immune defense and disease pathogenesis (21). Among chemokines, CXCL10 (IP-10) and CXCL8 (IL-8) demonstrated strong predictive performance, consistent with previous reports highlighting their role in leukocyte recruitment and granuloma formation (13). CCL2 also emerged as a key marker, supporting its known involvement in monocyte trafficking and TB pathogenesis. Chemokines are key immunoregulatory molecules that plays a critical role in disease progression by the recruitment and trafficking of immune cells, including monocytes, Dendritic cells, T lymphocyte and NK cells (14). CCL2 recruits monocytes, CXCL10 recruits activated T cells, and CXCL8 and CXCL1 promote neutrophil recruitment. Although these responses support host defence, excessive chemokine activity may cause inflammation, pulmonary damage, and unfavourable treatment outcomes (13). In our study we observed significantly elevated levels of CCl-2, CCL-4, CXCL-1, CXCL-8, and CXCL-10 in individuals with unfavourable treatment outcomes, indicating a heightened inflammatory and immune activation state. These findings suggest that dysregulated chemokine responses may contribute to impaired host control of infection and poor treatment response. Taken together, our results reinforce the growing evidence that specific chemokine signatures, particularly the combination of CCL-2, CXCL-8, and CXCL-10, hold promise as predictive biomarkers for treatment outcomes.

Matrix metalloproteinases, particularly MMP-2, MMP-7, and MMP-9, exhibited significant associations with unfavourable outcomes and showed good diagnostic performance both individually and in combination. These findings align with prior evidence indicating that MMPs contribute to extracellular matrix degradation, lung tissue damage, and cavity formation in TB (16, 18). The high AUC values observed for combined MMP panels further support their utility as markers of disease severity and progression. Our findings are consistent with previous studies, the elevated expression of these MMPs at baseline may reflect heightened immune activation and ongoing tissue remodeling, which could contribute to disease severity and unfavourable treatment outcomes (19, 22). MMPs are known to be regulated by inflammatory mediators, including cytokines and chemokines, thereby linking immune activation with structural tissue damage (19). Mechanistically, increased MMP activity may promote extracellular matrix degradation, pulmonary tissue destruction, and cavitation, potentially creating a pathological environment associated with persistent infection and delayed recovery. Thus, elevated MMP-2, MMP-7, and MMP-9 may reflect ongoing tissue remodelling and disease progression rather than protective immune activity alone (18). Taken together, the findings of this study underscore that cytokines, chemokines, and MMPs are significantly associated with unfavourable treatment outcomes. While individual biomarkers reflect distinct aspects of the host immune response, their combined assessment representing multiple immunological and pathological pathways enhances predictive accuracy and provides a more comprehensive understanding of disease progression.

A key strength of this study is the integration of advanced statistical and machine learning approaches, including ROC analysis, random forest-based feature importance, PCA, and stability selection. These analyses enabled the identification of a refined biomarker panel CXCL8, MMP-2, and CCL2 with high predictive accuracy (AUC ~0.9), demonstrating the advantage of multi-marker approaches over individual biomarkers. This is consistent with previous studies emphasizing that composite biosignatures provide improved prognostic performance in TB (13, 16).

Although our findings are consistent with previous studies, differences in the performance of individual biomarkers may reflect variations in study populations, disease severity, comorbidities, assay methods, and definitions of unfavourable outcomes. The improved performance of the combined CXCL8, MMP-2, and CCL2 panel supports the concept that TB treatment outcomes involve multiple biological pathways. Importantly, this study extends previous findings by validating these biomarkers in independent RePORT India cohorts. However, standardized assays and further external validation are required before clinical application.

These findings underscore the advantage of a multi-biomarker strategy for the early identification of patients at risk of poor treatment outcomes. Despite robust findings, this study is limited by reliance on a single regional external validation, necessitating further testing in diverse international cohorts to ensure generalizability. Additionally, evaluating biomarkers at only one time point prevents assessment of the dynamic fluctuations in chemokines and MMPs as treatment progresses. Future longitudinal studies are required to capture these critical immune changes over time. As this study utilized archived baseline samples from the RePORT India Consortium Central Biorepository, the retrospective use of available specimens may have limited control over participant selection and data completeness. Furthermore, because immune biomarkers were evaluated only at baseline, this study could not determine the temporal dynamics of these markers or identify the stage at which immune dysregulation begins to contribute to disease severity and unfavorable treatment outcomes. Therefore, longitudinal prospective studies with serial sample collection are needed to define the kinetics of these immune markers and establish their predictive value throughout the course of tuberculosis treatment and disease progression.

In conclusion, this study demonstrates that a combination of selected plasma chemokines and matrix metalloproteinases (MMPs), particularly CXCL-8, MMP-2, and CCL-2, holds promise as a predictive biomarker panel for adverse or unfavourable treatment responses. Various combinations of these markers consistently exhibited high diagnostic performance, with an AUC of 0.9 and well-balanced sensitivity and specificity. Overall, the study provides strong validation of host immune biomarkers and highlights a robust multi-marker panel for predicting unfavourable TB treatment outcomes. These results represent a significant step toward the development of clinically applicable prognostic tools, enabling improved risk stratification and more personalized management of TB patients.

## Data Availability

The original contributions presented in the study are included in the article/supplementary material. Further inquiries can be directed to the corresponding author.
